# Evaluating Burn First Aid Knowledge, Practices, and Confidence Levels Among the General Population in Aseer, Saudi Arabia

**DOI:** 10.7759/cureus.64760

**Published:** 2024-07-17

**Authors:** Mohammed E Elhussiny, Bandar M Abuageelah, Mona H Alfaifi, Mubarak M Alshahrani, Yousef M Alyami, Ghade T Aljaber, Halima A Alghamdi, Alhanouf F Banah, Maryam A Albaraq, Ohud A Alnaji, Alya I Majrashy, Hamzah M Alyami, Saifaleslam A Mahmoud, Khalid M Alameer

**Affiliations:** 1 Department of Histology, Faculty of Medicine, Al-Azhar University, Cairo, EGY; 2 Department of Histology, General Medicine Practice Program, Batterjee Medical College, Aseer, SAU; 3 General Medicine Practice Program, Batterjee Medical College, Aseer, SAU; 4 Faculty of Medicine, Jazan University, Jazan, SAU

**Keywords:** saudi arabia, public health, emergency, first-aid, burn

## Abstract

Background and objectives

Burns represents a significant public health issue globally and in Saudi Arabia, disproportionately affecting vulnerable groups. Prompt, evidence-based first aid improves outcomes. This study assessed burn first aid understanding, self-assurance, and information sources among Aseer Region residents.

Methods

A cross-sectional online survey was distributed to 386 individuals using a validated questionnaire, assessing understanding via a 10-item scale and confidence through Likert scales. Associations between variables were examined statistically.

Results

Most participants (85%; n=330) demonstrated poor first-aid comprehension, and only (1%; n=2) exhibited excellent knowledge. A history of burn exposure correlated with higher knowledge (p=0.039). The Internet was the primary information source (48%; n= 185). Confidence in assisting burn victims was generally low.

Conclusions

Significant gaps in foundational burn first aid knowledge were identified, necessitating targeted educational interventions disseminated via multiple modalities to strengthen emergency response and optimize outcomes in this region.

## Introduction

Burns refer to heat, chemical, electrical, or radiation-induced skin and soft tissue injuries. Common etiologies include scalds, flames, electricity, friction, and chemical contact. Such injuries pose serious risks by damaging skin and deeper tissues. Minimizing tissue injury severity and pain through prompt first aid application is paramount. Furthermore, immediate medical attention is crucial due to the potential for substantial physical, functional, occupational, cosmetic, and psychosocial ramifications [1,2].

Burn injuries pose a considerable burden to global health, though precise incidence data remains limited. The World Health Organization estimates over 265,000 annual burn-related deaths worldwide, underscoring burns as a driver of mortality and morbidity. Low-income communities in developing nations seem disproportionately impacted [3]. Within Saudi Arabia, burns represent a primary trauma etiology, particularly among young children often scalded by hot liquids. Mortality in pediatric patients is under 1% but rises to nearly 7% across all age groups [4]. Mitigating the occurrence of these potentially catastrophic injuries necessitates examining public awareness of burn prevention and first aid techniques.

Numerous survey-based studies conducted worldwide have assessed the awareness and attitudes of various populations regarding first aid. These studies consistently reveal a concerning lack of knowledge regarding first aid for burns, with many individuals resorting to unscientific topical remedies such as ice, herbal medicines, and various household items (e.g., oil, honey, vinegar, eggs, toothpaste, and flour). This improper first-aid approach often leads to an increase in post-burn complications [1,5]. Notably, research has shown that individuals who have undergone a first aid course tend to possess a better knowledge of burn first aid [6-8].

Few reports have explored the general population's awareness of and practices for initial burn care in Saudi Arabia. This study aims to evaluate burn first aid comprehension among residents of the Aseer region, particularly to identify priority areas for enhancement. The findings inform tailored educational initiatives and public messaging to improve the proper handling of burn emergencies in this region. Assessing first aid understanding and typical responses in Aseer offers opportunities to strengthen initial response and subsequent clinical care quality for burn injuries.

## Materials and methods

Study design, setting, and population

An observational cross-sectional online survey was performed using a pre-validated questionnaire [9,10]. This questionnaire was developed in electronic form using Google Forms and distributed among the general public of Aseer region via social media platforms. 

Sample size and study population

Based on data from the General Authority for Statistics in Saudi Arabia [11], the population of the Aseer region was estimated to be 1,444,688. Using the Raosoft sample size calculator (Raosoft Inc., Seattle, WA) and assuming a 95% confidence level and 5% margin of error, the minimum recommended sample size was calculated to be 385 individuals. Convenience sampling was employed to ensure practicality and accessibility in participant recruitment. Eligible participants included residents of Aseer aged 18 years or older. Those under 18 years and individuals who declined to participate were excluded.

Survey design and pilot study

Following established tools [9,10], a pre-existing, validated questionnaire originally developed in English was utilized. To ensure cultural appropriateness and comprehension, the questionnaire was translated into Arabic before being distributed to participants. To ensure content validity, expert review and adaptation from validated tools [9,10] were employed, and pilot testing was conducted with a sample of 10 participants to verify comprehension and linguistic accuracy in Arabic. The questionnaire's test-retest reliability was evaluated by administering it multiple times during the pilot phase and observing consistent responses. Through this iterative process, we arrived at the optimized form of the questionnaire.

Data collection

An online survey was developed using Google Forms to conveniently complete participants' questionnaires. The survey consisted of two sections. Section 1 was used to obtain the sociodemographic characteristics of the respondents. Section 2 evaluated burn first aid knowledge using 10 items to assess understanding related to burn management. Participants' confidence in administering burn first aid techniques was also gauged using a five-point Likert scale. Sources of information regarding burn first aid practices were also documented.

Statistical analysis

Survey responses were initially recorded in Microsoft Excel version 2016 spreadsheets. The collated data underwent statistical evaluation using SPSS 23.0 software (IBM Corp., Armonk, NY). The analysis included both descriptive and inferential components. Frequencies and percentages were computed and presented in tables for sociodemographic attributes and other categorical variables. Respondents' understanding of burn-related queries was evaluated using a 10-item scale, with one point allotted for each accurately addressed question. Scores were subsequently classified into three tiers of comprehension: Poor (less than 5 points), fair (5-7 points), and excellent (more than 7 points), as per previously validated methods [10]. Associations between categorical variables were examined using the chi-square test, with a significance level of p < 0.05 to indicate statistical significance.

Ethical approval

Participants voluntarily engaged in the questionnaire, ensuring the strict preservation of data confidentiality. Informed consent was digitally obtained from all participants, who were required to choose between "yes" or "no" after reviewing a consent form outlining the study's aims and procedures. This study received ethical approval from the Research Ethical Committee, Batterjee Medical College on March 12, 2023, under approval number RES-2023-0080.

## Results

A total of 386 individuals completed the survey (Table 1). According to the study participants' demographic data, 51% (n=196) of them were male, while 49% (n=190) were female; 60% (n=232) of the sample was between the ages of 18 and 29, and 84% (n=326) lived in urban areas. Most participants (48%, n=186) were students. University students made up 80% (n=310) of the study participants. Around 44% of the sample participants (n=168) made between 10,000 and 20,000 Saudi riyals monthly. Around 74% of the participants (n=284) had children under 18 living at home, and 69% (n=266) had not received formal training or education on burns and their management. Around 51% (n=197) had suffered burn injuries to themselves or close family members.

**Table 1:** A Comprehensive Overview of the Included Data (n = 386).

Variables		Frequency	Percent
Gender	Male	196	51%
	Female	190	49%
Age groups	18-29	232	60%
	30-49	140	36%
	> 50	14	4%
Residency	Urban	326	84%
	Rural	60	16%
Occupation	Student	186	48%
	Teacher	60	16%
	Military sector employee	18	5%
	Health sector employee	30	8%
	Unemployed	47	12%
	Retired	12	3%
	Housewife	11	3%
	Private sector	12	3%
	Other	10	3%
Educational level	Primary school	4	1%
	Intermediate school	4	1%
	Secondary school	68	18%
	University	310	80%
Household income	Less than 10,000 riyals (2,665$)	142	37%
	10,000 to 20,000 riyals (2,665$ - 5,331$)	168	44%
	From 21,000 to 30,000 riyals (5,597$ 7,996$)	35	9%
	More than 30,000 riyals (7,996$)	41	11%
With children/adolescents (under 18 years) living at home?	Yes	284	74%
	No	102	26%
Have you received any formal training/attended any workshop regarding burns and their management?	Yes	120	31%
	No	266	69%
History of exposure to burn injury (self or family member)?	Yes	197	51%
	No	189	49%

Table 2 displays the levels of knowledge on first aid management of burns among participants. The data has been divided into poor, fair, and excellent categories. Most of the participants (85%; n=330), showed a need for more knowledge in first aid for burns. Only two participants (1%) demonstrated an excellent understanding of managing burns through first aid.

**Table 2:** Knowledge Level Regarding First Aid Management of Burns of Participants (n = 386).

Knowledge Levels	Frequency	Percent
Poor (<50%)	330	85%
Fair (50-70%)	54	14%
Excellent (>70%)	2	1%

Table 3 explores associations between respondent characteristics and burn first aid knowledge levels. A statistically significant difference emerged based on the history of personal or familial burn exposure (p=0.039). Individuals with personal or familial burn exposure generally demonstrated higher comprehension than those without such experience. No other demographic variables, such as gender, age groups, residence location, training exposure, or occupation, yielded significant disparities in first aid knowledge. These findings suggest that direct contact with burn cases may confer modest advantages to familiarity with recommended practices.

**Table 3:** The Relationship Between Knowledge Regarding First Aid Management of Burns and Participant Variables (n = 386). *Statistically significant (p < 0.05).

Variables		Knowledge Levels						p-value
		Poor		Fair		Excellent		
		n	%	n	%	n	%	
Gender	Male	172	44.60%	23	6.00%	1	0.30%	0.43
	Female	158	40.90%	31	8.00%	1	0.30%	
Age groups	18-29	204	52.80%	27	7.00%	1	0.30%	0.548
	30-49	114	29.50%	25	6.50%	1	0.30%	
	>50	12	3.10%	2	0.50%	0	0.00%	
Residency	Urban	275	71.20%	50	13.00%	1	0.30%	0.088
	Rural	55	14.20%	4	1.00%	1	0.30%	
Are there children/adolescents (under 18 years) living at home?	Yes	240	62.20%	42	10.90%	2	0.50%	0.514
	No	90	23.30%	12	3.10%	0	0.00%	
Have you received any formal training/attended any workshops regarding burns and its management?	Yes	99	25.60%	21	5.40%	0	0.00%	0.27
	No	231	59.80%	33	8.50%	2	0.50%	
History of exposure to burn injury (self or family member)?	Yes	161	41.70%	34	8.80%	2	0.50%	0.039*
	No	169	43.80%	20	5.20%	0	0.00%	

**Figure 1:**
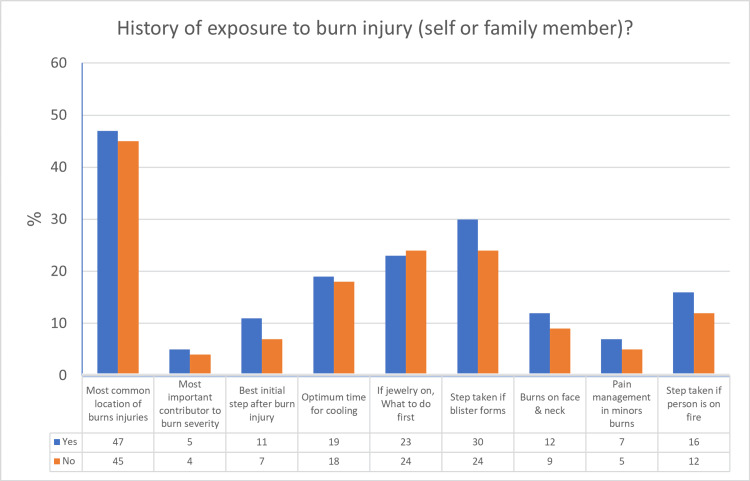
A Comparative Analysis of Response Accuracy Percentage Among Individuals With or Without Previous Exposure to Burn Injuries, Either Personally or Through Family (n = 386).

The study findings provide insight into the relationship between personal experience with burn injuries and a factual understanding of first aid protocols for burns. Respondents with a history of burn exposure, whether direct or involving close contacts, demonstrated higher rates of accurate responses across domains assessed regarding burn management. For instance, when queried about appropriate steps if blistering occurs, 30% (n=115) of those with prior experience selected the correct answer compared to 24% (n=92) of individuals without such exposure. Regarding the best initial action should someone's clothes be ablaze, 16% (n=61) of previously exposed participants responded correctly versus 12% (n=46) of those with no injury history. Similarly, 11% (n=41) of those with experience handling burn cases answered accurately for the best initial measure following any burn, compared to only 7% (n=27) without prior involvement. These differences in proportions of correct answers contingent on experience with burns are represented in Figure 1. The findings suggest that personal engagement with or witnessing burn incidents confer advantages to cognitive familiarity with recommended first aid practices.

**Figure 2:**
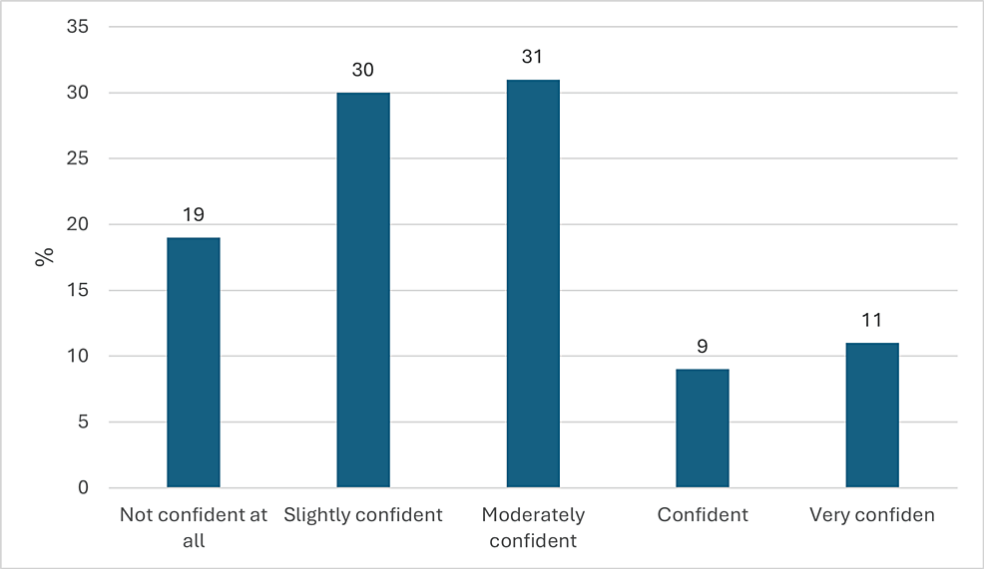
Assessing Individuals' Self-Assurance in Delivering First Aid to Burn Victims (n = 386).

Figure 2 indicates a prevalent lack of self-assurance within the general population concerning their proficiency in providing initial aid to individuals suffering burn injuries, regardless of their level of knowledge. More specifically, 31% (n=120) of respondents expressed being "moderately confident," "slightly confident," or "not confident at all," with corresponding percentages of 30% (n=116) and 19% (n=73), respectively.

**Figure 3:**
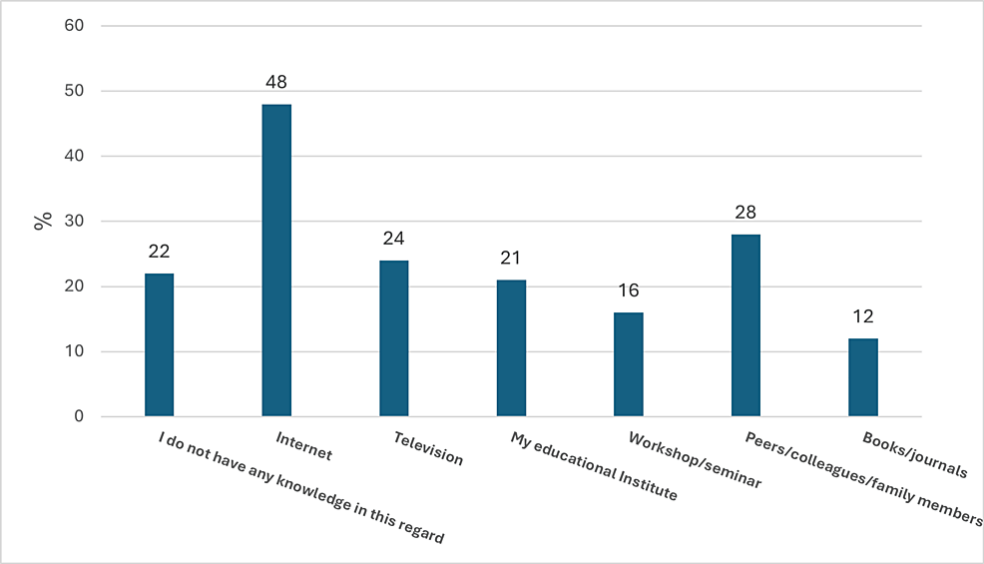
Exploring Knowledge Sources of First Aid Techniques for Burn Management (n = 386).

The study findings provide insight into the primary channels through which respondents acquired familiarity with burn first aid protocols. As shown in Figure 3, the Internet surfaced as the most widely accessed resource, cited by 48% (n=185) of participants. However, a sizeable proportion (22%, n=85) reported having no previous awareness of burn management techniques. The percentage of drawing knowledge from academic sources like books and journals was the lowest (12%, n=47).

## Discussion

The present study evaluated foundational knowledge of optimized burn first aid among Aseer Region residents. Previous epidemiological investigations in Saudi Arabia commonly implicated electrical injuries, flames, and scalds as chief etiologies, predominantly affecting males and children [12-14]. Timely delivery of evidence-based initial measures can favorably influence recovery [15,16]. Specifically, running cool water for 20 minutes, removing constrictive items (e.g., jewelry and clothing), and applying sterile covering are recommended [15,16]. Regarding re-epithelialization, scar attributes, and cosmetic outcomes, wound therapies involving 15°C and 2°C water generally yielded more favorable results versus others, while ice should be avoided [15].

The current study showed that most of the respondents were young with a high level of education. Also, nearly one-third of them received formal training or attended workshops regarding burns and their management, and nearly half of them were exposed to burn injury (self or family member).

This study revealed generally insufficient foundational knowledge of burn first aid among Aseer Region residents, with the Internet cited most commonly as an information source rather than clinical staff, books/journals, or workshops. This likely contributes to the observed poor levels of comprehension. Participants demonstrated the highest level of awareness regarding typical burn locations and blister care steps, while their knowledge regarding pain management and factors affecting severity showed the lowest rates. This aligns with findings from Asiri et al., who reported that 73.6% of participants exhibited inadequate first-aid knowledge for burns, with only 26.4% adequately informed. The most prevalent misconception endorsed applying toothpaste, honey, or ice to burns, which are not recommended treatments [17]. Overall, results indicate the need for targeted educational interventions delivered via multiple modalities to optimize emergency response.

The present study identified scalds from hot water as the most reported cause of burns. A similar knowledge level was assessed by Hsiao et al. [18], who found that only 36% of participants knew how to treat burns with first aid, 13% said they would cool the burn area with water, and 7% said they could put out a fire by rolling on the ground. However, many students said they would apply toothpaste (18%), treat burn injuries right away without allowing them to cool down, or just phone for assistance and do nothing. Similarly, Davies et al. found that 32% demonstrated sufficient first-aid knowledge, irrespective of demographics (e.g., age, social status, or income) [19]. In Vietnam, Quynh et al. [20] found that about 60% of the participants lacked knowledge about thermal injury first aid, 38.7% would apply cool fresh water, 15% would bandage the burn site temporarily, and 80.5% would leave the blister intact. The media came in third with 36% of the information, followed by friends, family, and other relations with 46%. Regarding burn first aid comprehension within Saudi Arabia, Al-Qahtani et al. reported more optimized knowledge among participants to some degree [9]. Specifically, the majority, at 78.5%, recognized not applying raw eggs or herbs directly to wounds. Additionally, 82.6% knew the importance of stopping, dropping, and rolling if clothing ignites. While still demonstrating room for enhancement, over 40% endorsed cleaning hot oil hand injuries with cold water (43.8%) or seeking hospital care for burns (41.0%).

The literature provides additional insights into burn first aid comprehension internationally. Harvey et al. administered a telephone survey in New South Wales, finding that 82% of participants endorsed water for cooling, but only 9.4% specified the recommended 20-minute timeframe. Less than 1% recognized alternative validated procedures [8]. In the USA, Taira et al. interviewed burn victims about their prehospital care and discovered 73% cooled injuries, with 39.9% using tap water, 25.2% ice, 8.9% cooling blankets, and 22.2% applying dressings alone. Though endorsement of cooling exceeded 50% in both investigations, adherence to evidence-based protocols appeared suboptimal [21]. Wallace et al. administered surveys assessing burn first aid knowledge, finding satisfactory comprehension ranged from 30-50% dependent on etiology. Participants receiving training within five years answered correctly, around 15% more often [6]. Comparably, the present study associated higher knowledge levels with personal/familial burn experience or rural residence to some degree. More than three-fourths expressed confidence in providing first aid, though few reported high confidence. Other investigations have similarly reported low preparedness and suboptimal practices globally regarding burn management [22-24]. Collectively, these studies highlight targeted educational programming as an ongoing priority. Standardizing minimum first aid protocols could help optimize frontline response capacity worldwide while reducing variability. Future research evaluating tailored training modalities may offer insights to strengthen the translation of guidelines into practical competencies.

The present study faces certain methodological limitations. Firstly, using a convenience sampling approach may result in a sample that does not sufficiently represent the target population, thus limiting the generalizability of our findings. Secondly, relying solely on self-reported data collected through online questionnaires introduces the possibility of reporting biases if participants provide inaccurate or incomplete responses. Variable comprehension of questions may also affect data quality. Additionally, online data collection without direct supervision may compromise validity and reliability if respondents' environments are uncontrolled. Excluding individuals without internet access introduces selection bias. Finally, while piloted, the questionnaire may not identify all potential issues impacting accuracy or validity.

## Conclusions

The current study revealed that most participants had poor knowledge and awareness about burns and their associated first-aid protocols. A history of direct experience with burn injuries was linked to enhanced understanding. Furthermore, participants generally lacked self-assurance in managing burn cases, likely driven by deficiencies in knowledge. Critically, these findings validate the necessity of implementing a targeted educational initiative for the general population and significantly advancing healthcare providers' familiarity and grasp of burn first aid principles. Optimizing clinicians' learning represents a promising strategy for improving burn patient outcomes and care delivery. With enhanced preparation through focused training programs, the population's response to burn emergencies stands to benefit substantially. Overall, the study reinforces the importance of public health efforts to close prevailing knowledge gaps through innovative awareness campaigns and hands-on instructional methods.
